# Fast DNA Vaccination Strategy Elicits a Stronger Immune Response Dependent on CD8^+^CD11c^+^ Cell Accumulation

**DOI:** 10.3389/fonc.2021.752444

**Published:** 2021-12-07

**Authors:** Chenlu Liu, Xianling Cong, Yuqian Wang, Qianqian Guo, Yu Xie, Fei Geng, Jie Guo, Ling Dong, Yi Zhou, Hui Wu, Bin Yu, Jiaxin Wu, Haihong Zhang, Xianghui Yu, Wei Kong

**Affiliations:** ^1^ National Engineering Laboratory for AIDS Vaccine, College of Life Science, Jilin University, Changchun, China; ^2^ Biobank, China-Japan Union Hospital of Jilin University, Jilin University, Changchun, China; ^3^ Key Laboratory for Molecular Enzymology and Engineering, College of Life Science, Jilin University, Changchun, China

**Keywords:** DNA vaccine, short-interval vaccination strategy, antigen-presenting cells, chemokines, lung cancer

## Abstract

Conventional DNA vaccine strategies usually employ a regimen of immunizations at 2-week or longer intervals to induce effective memory cell-dependent immune responses. Clinical cancer treatment requires a faster immunization strategy to contend with tumor progression. In this study, a novel fast immunization strategy was established, wherein a DNA vaccine was intramuscularly administered on days 0, 2, and 5 in a murine lung cancer model. Effector cells peaked 7 to 10 days after the last vaccination. Compared with traditional 2-week-interval immunization strategies, antigen-specific cytolysis and INF-γ secretion were significantly enhanced under the fast vaccination approach. As a result, the rapidly administered DNA vaccine elicited stronger and more prompt antitumor effects. The probable underlying mechanism of fast immunization was the accumulation of CD8^+^CD11c^+^ antigen-presenting cells at the injection site, which enhanced subsequent antigen presentation. In conclusion, the fast DNA vaccination strategy shortened vaccination time to 5 days and elicited a stronger antitumor immune response.

## Introduction

DNA vaccines are a promising cancer immunotherapeutic strategy, offering advantages such as a favorable safety profile, and simple preparation and storage requirements (1, 2). Additionally, DNA vaccine also has an advantage in inducing CD8^+^ and CD4^+^ T cell response, which is responsible for the clearance of tumor cells (3). However, given the insufficient immunity induced by a single dose of DNA vaccines, immunization requires the administration of several doses. In the current vaccination strategies, an interval of at least 2 weeks is recommended between doses, thus requiring at least a month or more for the generation of a robust immune response (4–6). Therefore, immune response induction *via* DNA vaccine therapy is generally considered time-consuming (7, 8). This slow process struggles to effectively counteract rapid cancer progression (9). To date, a fast DNA vaccination strategy that can efficiently elicit immune responses has not been established.

Recently, a personalized poly-epitope DNA vaccine of Washington University School of Medicine is in Phage I clinical trials among triple-negative breast cancers (NCT02348320). According to statistics in ClinicalTrial.gov, accumulating cancer DNA vaccines are in Phage I or II clinical trials (such as NCT02204098, NCT04090528, and NCT03600350). DNA vaccine has developed in many fields. The antigens have changed from traditional tumor-specific antigens or tumor-associating antigens to neoantigens (NCT03122106) (10). The delivery system has also developed to enhance the immunogenicity. Gene gun, electroporation, Tattoo, and many other devices are applied in DNA vaccine (7, 11). However, most of DNA vaccines are still vaccinated with several-week intervals. The vaccination strategy may be the limitation of DNA vaccine in the future. Most cancer vaccines, with the exception of those targeting virus-induced tumors, are designed as therapeutic vaccines (12–14). Prophylactic vaccine employs this strategy and is vaccinated with 2 or more-week intervals. Memory B or T cells could be generated after several vaccinations and protect hosts from being infected (8, 9, 15). This process of memory cell generation is time-consuming. When it is applied in therapeutic cancer vaccine, this strategy showed insufficient. A single dose of DNA vaccine could not induce strong enough antigen-specific effector cells or memory lymphocytes to control the rapid cancer progression (16). The rapid nature of cancer progression highlights the need for more expeditious strategies for therapeutic cancer vaccination.

Schumacher and colleagues previously reported a fast DNA vaccination strategy, wherein vaccines were administered three times in 1 week and elicited a stronger immune response due to prolonged antigen expression in subcutaneous tissue. However, in this strategy, the vaccines were to be administered intradermally, as no enhanced effects were observed after intramuscular injection (7). Administration route-associated restrictions may thus limit the application and promotion of fast vaccination strategies.

Intramuscular injection is most frequently used for DNA vaccine administration (17–19). While various muscle cells express antigens encoded by the administered DNA, a lack of antigen-presenting cells (APCs), such as dendritic cells (DCs), has been reported at the site of intramuscular injections (20, 21). Thus, enhancing APC infiltration into the muscle injection site may be an effective method for the rapid induction of a potent immune response (21, 22).

CD11c is a transmembrane integrin alpha X chain protein highly expressed on DCs, most monocytes/macrophages, some B cells, and natural killer cells. In peripheral muscle tissue, DCs and macrophages—both APCs—express CD11c. DCs are the major APCs for T lymphocyte activation (23, 24). The cross-presentation of antigens encoded by DNA vaccines is critical for the induction CD8^+^ T cell immunity (24, 25). The migration of APCs to the inflamed vaccine injection site is facilitated by chemokines (26, 27). Depending on the vaccine adjuvant, DCs could be found to accumulate at the muscle injection site (28–30). In the current study, we sought to determine whether the DNA vaccine-induced immune response could be potentiated by increasing APC recruitment to the injection site. Hence, we analyzed the expression of chemokines and the abundance of APCs at the site of injection after vaccination. Based on the timing of APC migration, we designed a fast vaccination strategy. We used the well-studied CpDV-IL2-sPD1/MS DNA vaccine, which encodes two tumor antigens, MUC1 (M) and survivin (S), and included soluble PD1, CpG motif, and IL-2 as immunoadjuvants to enhance the CD8^+^ T cell-mediated antitumor immune response. Genes of MUC1 and survivin (MS) were fused to express in vaccine. Soluble PD1 (sPD1) interacts with PD-L1 on the surface of DCs to improve antigen uptake and presentation. CpG is a TLR9 agonist, and IL-2 is a major Th1 cytokine; both molecules play an important role in lymphocyte activation, as demonstrated in our previous research (31, 32).

CD8^+^ effector T cells are the mainly executors of killing tumor cells. Hence, to induce a strong enough immune response as soon as possible will improve the antitumor (16). Therefore, we hypothesize that fast vaccination strategies should perform better than currently applied DNA vaccine administration regimens. In this study, we compared the antitumor efficacy of fast DNA vaccination with that of conventional strategies and sought to elucidate the molecular mechanism underlying the efficacy of the fast vaccination approach. We postulated that a fast vaccination strategy would allow for the recruitment of APCs to the injection site. In order to further enhance the immune response and antitumor effect of fast immunization using a DNA vaccine, we use the DNA primed-recombinant adenovirus (rAd) vaccine boost strategy, which was demonstrated to be an effective antitumor strategy in our previous study (33). The current findings highlight the potential of a novel fast DNA vaccination strategy that merits further evaluation in preclinical and clinical research.

## Materials and Methods

### Mice

Experiments with C57BL/6 and BALB/c mice (Beijing Huafukang Biology Technology Co., Ltd., China) were approved by the Ethical Committee for the Use of Laboratory Animals at Jilin University and were carried out in strict accordance with the National Institutes of Health Guide for the Care and Use of Laboratory Animals. Mice were female, 6 to 8 weeks old.

### Cell Line

Mouse colorectal cancer cell line CT26 was preserved at the National Engineering Laboratory for AIDS Vaccine of Jilin University. Mouse lung cancer cell line Lewis (No.: GF123) was purchased from Shanghai Gefan Biotechnology Co., Ltd. Lewis-GFP-MS and CT26-GFP-MS cell lines stably expressing tumor antigens MUC1 and survivin were previously generated in our laboratory (31).

### Plasmid and Virus Sample Processing

The DNA vaccine CpDV-IL2-sPD1/MS and rAd, Ad-MS, were previously constructed (31, 33). DNA vaccine plasmids were amplified and purified by Changchun BCHT Biotechnology Co. The rAd vaccine was manufactured by Shenzhen Yuanxing Gene Technology Co., Ltd., and contained 2 × 10^11^ vp/ml. Both vaccines were diluted to the final concentration in PBS (phosphate-buffered saline) buffer.

### Immunization and Tumor Challenge

Two immunization strategies were used in this research based on vaccination intervals, namely, a traditional immune strategy (normal immunization strategy) with 1- or 2-week intervals, and a fast immunization strategy (days 0–2–5) with 2-day and 3-day intervals. Both DNA and rAd vaccines were administered intramuscularly. DNA vaccine is vaccinated at a dosage of 100 µg or a triple dosage.

For tumor inoculation, C57BL/6 mice were subcutaneously injected with 1.3~1.4 × 10^5^ Lewis-GFP-MS cells in the right hind flank, and the day of inoculation was recorded as day 0. In the normal immunization strategy group, the DNA vaccine was administered on day 1. In the fast immunization group, the DNA vaccine was administered when the tumor reached approximately 50~100 mm^3^, which occurred between day 4 and day 8. Tumor size was measured using a caliper, and the tumor volume was calculated (1/2 length × width^2^). The average volume in control group mice on the day of treatment initiation was set to a value of 100% (34, 35). Tumor-bearing mice were euthanized when the maximum diameter measured over 1.5 cm.

### Quantitative Real-Time PCR

Total RNA of muscle or inguinal lymph nodes samples was isolated using TRIzol reagent (Invitrogen). cDNA was synthesized, and qRT-PCR was then performed to analyze the expression of chemokine genes as previously described. The quantitative real-time PCR (qRT-PCR) primer sequences were as follows: *β-actin* forward 5′-CAAGCAGGAGTACGACGAGT-3′, reverse 5′-GGCTGGCATGAGGTGTGTA-3′; *Ccl3* forward 5′-CCTCTGTCACCTGCTCAACA-3′, reverse 5′-GATGAATTGGCGTGGAATCT-3′; *Ccl4* forward 5′-CCCACTTCCTGCTGTTTCTC-3′, reverse 5′-GAGGAGGCCTCTCCTGAAGT-3′; *Ccl5* forward 5′-TGCCCACGTCAAGGAGTATTTC-3′, reverse 5′-AACCCACTTCTTCTCTGGG TTG-3′; *Ccl19* forward 5′-CACTCACTCTCTGTGGCCT-3′, reverse 5′-GGGCCAGAGTGATTCACATC-3′; *Ccl20* forward 5′-TGCTCTTCCTTGCTTTGGCATGGGTA-3′, reverse 5′-TCTGTGCAGTGATGTGCAGGTGAAGC-3′; and *Ccl21* forward 5′-TACTGGGCTATCCTCTTGA-3′, reverse 5′-ATGGCTCAGATGATGACTCT-3′.

### Preparation of Cell Suspensions From Spleen, Muscle, and Tumor Tissues

The spleen was dissected, washed with sterile PBS buffer, cut into pieces, ground, and filtered with gauze twice to remove connective tissue. Red blood cells were removed using red blood cell lysis buffer (ACK, BioLegend, San Diego, CA, USA). Cellular debris were removed *via* centrifugation. The concentration of splenocyte suspensions was adjusted to 1 × 10^7^ cells/ml.

Tumor or muscle cell suspensions were prepared in a similar manner. However, prior to tissue grinding, tumor or muscle tissues were cut into pieces and incubated with collagenase I (final concentration: 40 μg/ml, Roche, Basel, Switzerland) for 2 h at 37°C and 5% CO_2_. The tumor tissues were subjected to the same procedures as described for splenocyte suspensions.

### IFN-γ Secretion

IFN-γ released by splenocytes following MUC1- and survivin-specific stimulation was detected using an ELISpot kit as previously described. The peptide mixture, including survivin (H2-K^d^ sequence for BALB/c mice: AFLSVKKQF and H2-D^b^ sequence for C57BL/6 mice: STFKNWPFL, final concentration: 10 μg/ml) and MUC1 (H2-K^d^ sequence: APDTRPAPG and H2-D^b^ sequence: SAPDTRPAP, final concentration: 10 μg/ml), was used to stimulate splenocytes.

### Cytotoxic T Lymphocyte Assay

We used carboxyfluorescein diacetate succinimidyl ester (CFSE) staining to evaluate splenocyte cytotoxicity. Lewis-GFP-MS cells and wild-type Lewis cells were stained with high (5 μM) and low (0.5 μM) concentrations of CFSE. Splenocytes from C57BL/6 mice and the target Lewis-GFP-MS/Lewis cells were incubated at different effector-to-target (E:T) ratios for 8~10 h at 37°C and 5% CO_2_, followed by analysis of dead target cell percentage among Lewis-GFP-MS cell and wild-type Lewis cell populations using a FACS Caliber instrument (BD Biosciences). Splenocytes from BALB/c mice were incubated with CT26-GFP-MS/CT26 treated with CFSE as described above. Lewis-GFP-MS/Lewis or CT26-GFP-MS/CT26 was determined by the strains of mice. Cytotoxicity activity was detected by flow cytometry, and specific lysis was calculated as follows: specific lysis (%) = [1 - (peptide-loaded cells/unloaded cells from the experiment group)/(peptide-loaded cells/unloaded cells from target cell control group)] × 100 (36, 37).

### Isolation of CD11c^+^ Cells

CD11c^+^ cells were isolated from different tissues using the CD11c MicroBeads UltraPure mouse kit (Miltenyi Biotec, Order no. 130-108-338) with an LS-positive selection column, as per the manufacturer’s instructions. Muscle tissues were cut into pieces and incubated with collagenase for 2 h at 37°C and 5% CO_2_. A total of 10^8^ cells were resuspended in 400 µl buffer. CD11c Microbeads UltraPure (100 µl) were then added and incubated for 10 min in the dark at 2°C-8°C, followed by separation using the appropriate columns.

### Flow Cytometry (FACS)

The frequency of different immune cell types was determined using FACS. Myeloid-derived suppressor cells (MDSCs) in tumor or spleen tissue were analyzed using a mouse MDSC Flow Cocktail 1 (CD11b PE/Gr-1 APC/Ly-6G FITC, BioLegend). Regulatory T cells were analyzed using the Mouse Regulatory T Cell Staining Kit #1 (eBioscience). FITC anti-mouse CD8α antibody (clone: 53-6.7, BioLegend), APC anti-mouse CCR5 antibody (clone: HM-CCR5, BioLegend), PE anti-mouse CCR6 antibody (clone: 29-2L17, BioLegend), and PE/Cy7 anti-mouse CCR7 antibody (clone: 4B12, BioLegend) were used to label isolated CD11c^+^ cells (38).

### Statistical Analysis

All statistical analyses were performed with GraphPad Prism software (version 7.0, GraphPad, Inc., La Jolla, CA, USA) using unpaired t-tests and One-way ANOVA, as previously described.

## Results

### Upregulation of Chemokines at the Injection Site and Ipsilateral Inguinal Lymph Nodes After DNA Vaccination

**Figure 1:**
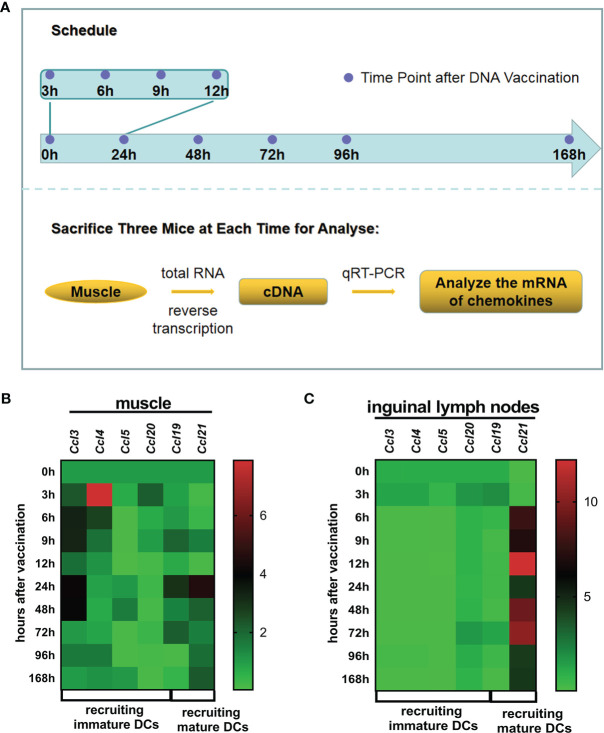
Chemokines within muscle at the injection site and ipsilateral lymph nodes. **(A)** Administration and treatment schedule. Twenty-seven BALB/c mice were vaccinated with 100 μg CpDV-IL2-sPD1/MS DNA vaccine intramuscularly in hindlimb skeletal muscle. At 0, 3, 6, 9, 12, 48, 72, 96, and 120 h after the injection, three mice were sacrificed, and the muscle at the injection site and the inguinal lymph nodes were dissected. Total RNA was extracted and reverse-transcribed to cDNA. Quantitative real-time PCR (qRT-PCR) was conducted to analyze chemokine mRNA levels. The relative mRNA levels of chemokines recruiting immature dendritic cells (DCs) (*Ccl3*, *Ccl4*, *Ccl5*, and *Ccl20*) and those recruiting mature DCs (*Ccl19* and *Ccl21*) in muscle at the injection site **(B)** and ipsilateral lymph nodes **(C)**.

APC migration is driven by chemokines. To define which specific chemokines were upregulated after the first vaccination, we analyzed chemokine mRNA expression. The upregulation of a series of chemokines was observed in muscle or the ipsilateral inguinal lymph nodes throughout 0–168 h (time points: 0, 3, 6, 9, 12, 24, 48, 72, 96, and 168 h) after DNA vaccine administration (**Figure 1A**). The mRNA levels of *Ccl3*/*Ccl4*/*Ccl5*/*Ccl20* (recruiting immature DCs, iDCs) and *Ccl 19*/*Ccl 21* (recruiting mature DCs, mDCs) were determined (**Figure 1**). At the injection site, *Ccl3* was upregulated approximately 3.88~4.11-fold during 24~48 h post-vaccination, *Ccl5* expression increased 1.57-fold at 48 h post-vaccination, and *Ccl*4 temporarily increased about 8-fold at 3 h post-vaccination (**Figure 1B**). *Ccl19* increased 4.56-fold at 24 h, while *Ccl21* increased 2.14~4.56 fold during 24~48 h (**Figure 1B**). When APCs uptake antigen at the injection site, they could carry the vaccine or antigen to lymph node acting the call for CCL19 or CCL21 (15, 26, 27). Hence, these chemokines mRNA were also analyzed in lymph nodes. Within the ipsilateral inguinal lymph nodes, of iDC-recruiting chemokines *Ccl3/Ccl4/Ccl5/Ccl20*, only *Ccl20* exhibited a modest 1.294-fold increase at the 72-h time point, while low expression was observed for the rest (**Figure 1C**). mDC-recruiting *Ccl21* was considerably upregulated 12.21-fold at the 9-h point, 9.30-fold at the 48-h point, 10.24-fold at the 72-h point, and 4.38-fold at the 96-h point (**Figure 1C**). These results suggested that local proinflammatory chemokines were upregulated at the injection site after DNA vaccination, which could enhance the recruitment of APCs, particularly DCs, and *Ccl21* began to be upregulated in lymph nodes since 24 h later DNA vaccination.

### Migration of CD11c^+^ APCs Into the Injection Site and Homing to Ipsilateral Inguinal Lymph Nodes

**Figure 2:**
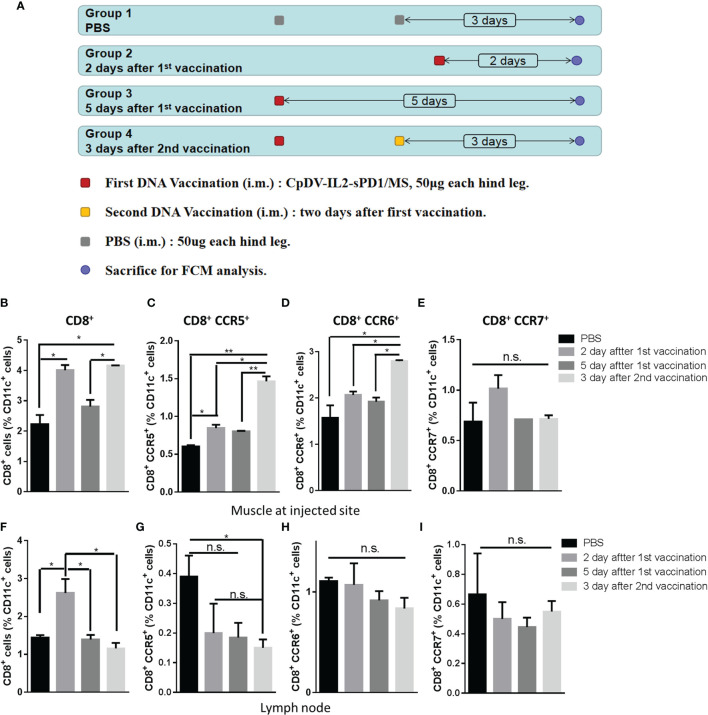
Timeline of CD11c^+^ antigen-presenting cell (APC) migration to the injection site and homing to ipsilateral inguinal lymph nodes. **(A)** Four groups of BALB/c mice (three mice in each group) were vaccinated as indicated, and 100 μg CpDV-IL2-sPD1/MS was injected intramuscularly at each time point. All mice were sacrificed on the same day. CD11c^+^ cells were isolated and analyzed *via* flow cytometry. Percentages of CD8^+^, CD8^+^/CCR5^+^, CD8^+^/CCR6^+^, and CD8^+^/CCR7^+^ subsets among CD11c^+^ cells isolated and enriched from muscle cells **(B–E)** and lymph nodes **(F–I)**. (*p < 0.05, **p < 0.01, n.s., p > 0.05).

CCR5 is the receptor of CCL3, CCL4, and CCL5, CCR6 is the receptor of CCL20, and CCR7 is the receptor of CCL19 and CCL20 (39). CCR5 and CCR6 could express on immature DCs and macrophage, interact with CCL3, CCL4, CCL5, and CCL20, and migrate APCs to the inflammatory site, including DNA vaccine injection site. When APCs internalize the vaccine, APCs downregulate CCR5 and CCR6, upregulate CCR7, and chemotactically migrate to lymph nodes by CCL19 and CCL21. Antigen presentation by APCs is a key process for immune response induction. CD11c is a prevalent APC marker (40, 41). To validate whether chemokines could recruit APCs into the injection site, CD11c^+^ cells were isolated from the injection site and ipsilateral inguinal lymph nodes at different time points after the first or second vaccination and analyzed *via* FACS. BALB/c mice were divided into four groups, and the immunization process is shown in **Figure 2A**. Two days after the first vaccination, we observed a nearly twofold increase of CD8^+^CD11c^+^ cell accumulation at the injection site. Five days post-vaccination, the number of CD8^+^CD11c^+^ cells decreased back to that of the PBS group. Upon the second vaccination 2 days after the first, CD8^+^CD11c^+^ cells accumulated back to previously observed levels after 3 days (**Figure 2B**). These results indicated that CD11c^+^ APCs were recruited to the injection site post-vaccination.

Chemokine receptor expression was also analyzed to determine CD11c^+^ cell migration. CD8^+^CCR5^+^CD11c^+^ cells, responding to CCL3, CCL5, and CCL20, exhibited a similar migration pattern to that of CD8^+^CD11c^+^ cells and were significantly recruited to the injection site post-vaccination (*p* < 0.05) (**Figure 2C**). CD8^+^CCR6^+^CD11c^+^ cells, also responding to CCL3, CCL5, and CCL20, were observed at the injection site after the second vaccination (*p* < 0.05) (**Figure 2D**). In addition, CCR7^+^ cells responding to CCL21, a marker of mDCs, exhibited a modest increase (*p* > 0.05) 2 days after the first vaccination, but no significant differences were observed between the four groups (**Figure 2E**).

CD11c^+^ cells migrated away from the injection site 5 days after the last vaccination in each group. Thereafter, we investigated whether these cells were attracted to the ipsilateral inguinal gland of the injection site. However, only CD8^+^CD11c^+^ cells appeared significantly increased two days after first injections (*p* < 0.05) (**Figure 2F**). iDCs expressing CCR5 or CCR6 exhibited a decline in ipsilateral inguinal gland (**Figures 2G, H**). No change was observed for CCR7^+^CD11c^+^ mDCs (**Figure 2I**).

Data in **Figure 2** indicate that CD11c^+^ APCs may accumulate at the injection site 2 days after the first vaccination as well as after the administration of booster vaccines.

### Fast DNA Vaccination Elicited Stronger Immune Responses

**Figure 3:**
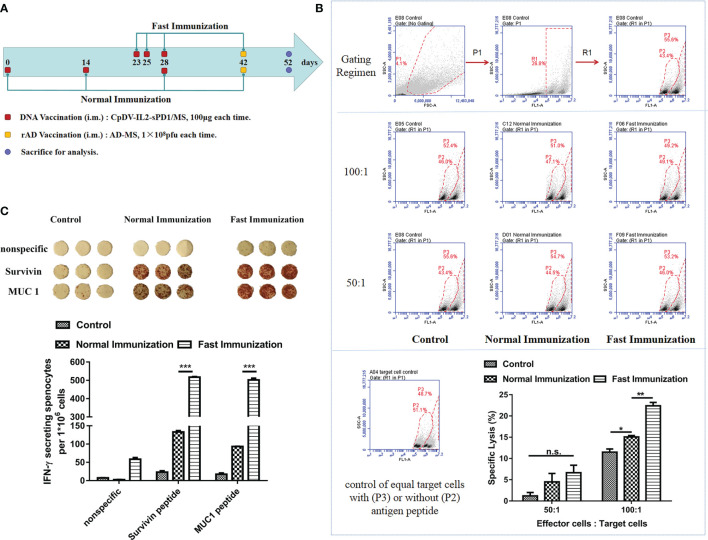
The fast immunization strategy elicited stronger antigen-specific immune responses than did the normal immunization strategy. **(A)** Immunization regimen in BALB/c mice. Three groups of BALB/c mice (five mice per group) were vaccinated intramuscularly with CpDV-IL2-sPD1/MS (100 µg in 100 µl) or PBS (negative control) three times. DNA vaccines were administered at 2-week intervals in the normal immunization group or on days 23, 25, and 28 in the fast immunization group. Recombinant adenovirus (rAd) (1 × 10^8^ *pfu* in 100 µl per mouse) was administered 2 weeks after the last vaccination (day 42) in both groups. PBS was administered under the fast immunization regimen as a negative control. Ten days after rAd vaccination (day 52), all mice were sacrificed to evaluate the immune response. **(B)** CTL assay. CT26 and CT26-GFP-MS were labeled with carboxyfluorescein diacetate succinimidyl ester (CFSE) of different concentrations (0.5 μM and 5 μM). Splenocytes and a mixture of target cells were incubated at ratios of 50:1 and 100:1, respectively, for 8 h at 37°C, 5% CO_2_. The percentage of cell death was determined *via* flow cytometry. Gating for CT26 and CT26-GFP-MS target cells treated with different CFSE concentrations was performed (upper panel). The percentage of CT26-GFP-MS (P3 Gate) and CT26 (P2 Gate) are shown (middle panel). The percentage of specific cytolysis was calculated (lower panel) **(C)** ELISpot assay. Splenocytes were incubated with hSurvivin peptide (H2-K^d^ sequence: AFLSVKKQF, final concentration: 10 µg/ml) and hMUC1 peptide (H2-K^d^ sequence: APDTRPAPG, final concentration: 10 µg/ml), and the secretion of IFN-γ per million splenocytes (ELISpot assay) was detected. (n.s., p > 0.05, *p < 0.05, **p < 0.01, ***p < 0.001).

Based on the timing of CD11c^+^ cell accumulation at the injection site (**Figures 1**, **2**), we speculated that vaccination at days 0, 2, and 5 would induce a more rapid and pronounced immune response. To assess this, healthy BALB/c mice were divided into three groups and vaccinated following fast or conventional strategies and sacrificed to then evaluate antitumor immune responses (**Figure 3A**). According to our previous results, administration of DNA vaccine CpDV-IL2-sPD1/MS under the normal immunization strategy could only induce a modest immune response. Further, rAd could boost this immune response. Thus, we questioned whether the rAd boost could further enhance the immune response under fast vaccination. Hence, for this experiment, both normal and fast DNA vaccination were boosted with 2-week-interval rAd vaccination. To analyze the antigen-specific cytolysis ability of splenocytes (effector cells), cancer cells CT26 with or without antigens (targets cells) labeled with different concentrations of CFSE were co-incubated with splenocytes with a ratio (effector cells: targets cells) of 50:1 or 100:1. FACS was used to detect the antigen-specific lysis. Cytotoxic T lymphocyte (CTL) assay results revealed that splenocytes from the fast vaccination group killed 50% more tumor cells than those of the normal vaccination group (*p* < 0.01, **Figure 3B**). Further, the secretion of IFN-γ was 4~5-fold greater in the fast vaccination group compared with that in the normal vaccination group (*p* < 0.001, **Figure 3C**).

### The DNA Vaccine-Induced Antigen Accumulation at the Injection Site Did Not Enhance Immune Response Under Fast Vaccination

We designed the fast vaccination strategy based on the timing of APC migration. Fast vaccination induced a stronger immune response than normal vaccination (**Figure 3**). Moreover, as short-interval vaccination may cause prolonged antigen expression at the injection site, we questioned whether the fast vaccination strategy could also enhance the immune response *via* the accumulation of DNA vaccine-encoded antigens at the injection site.

**Figure 4:**
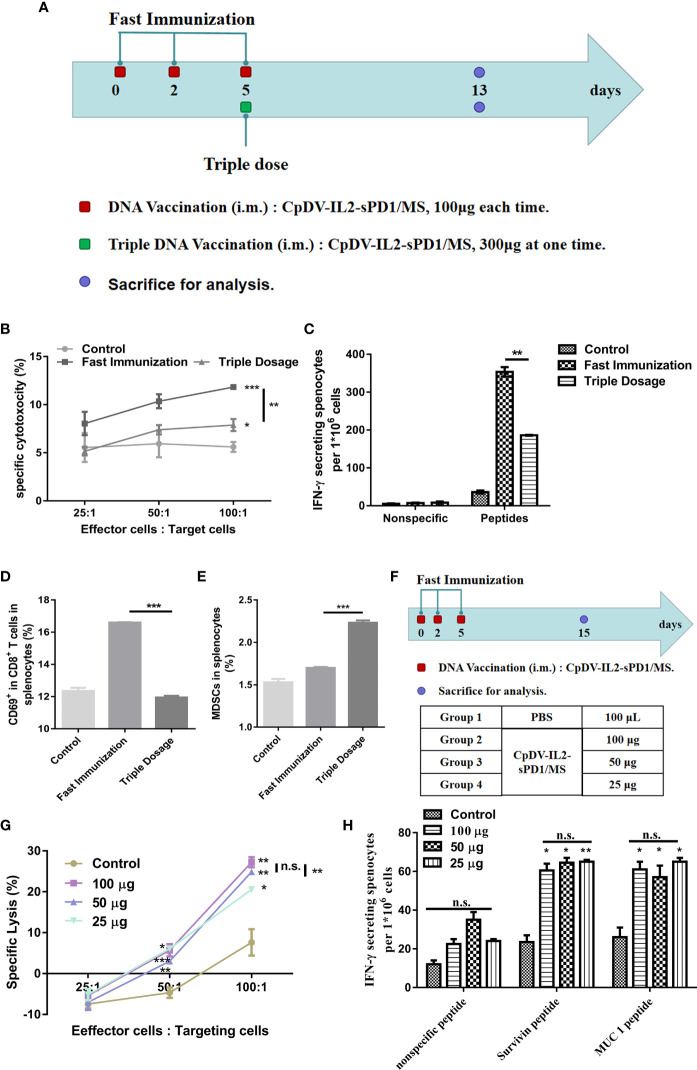
Efficacy of DNA vaccine dosage in the fast immunization strategy. **(A)** Immunization regimen. In the fast immunization group, BALB/c mice (n = 5) were vaccinated with 100 μg DNA vaccine on days 0, 2, and 5. In the triple-dosage group, mice were vaccinated with 300 μg DNA vaccine (150 μg in each thigh muscle) once on day 5. All mice were sacrificed on day 13. The splenocytes were separated and analyzed. **(B)** Cytotoxic T lymphocyte (CTL) assay. The E/T ratio was 25:1, 50:1, and 100:1, respectively. **(C)** ELISpot assay. Splenocytes were stimulated with peptides (survivin H2-K^d^ sequence: AFLSVKKQF, final concentration: 10 μg/ml; MUC1 H2-K^d^ sequence: APDTRPAPG, final concentration: 10 μg/ml). **(D)** Activation of CD8^+^ T cells. The percentage of CD69^+^-PE cells was determined by flow cytometry (FACS) with gating for CD3^+^FITC/CD8^+^ antigen-presenting cells. **(E)** Myeloid-derived suppressor cells (MDSCs) in lymphocytes. The percentage of MDSCs, CD11b- PE/Gr-1 APC/Ly-6G FITC, was detected using FACS. **(F)** Twenty BALB/c mice (n = 5) were divided into four groups. Vaccine doses included 25, 50, and 100 μg at each time point in the respective treatment group. The vaccination and intervals were as for the fast immunization strategy in **(A)**. **(G)** CTL assay. **(H)** ELISpot assay. (*p < 0.05, **p < 0.01, ***p < 0.001, n.s., p > 0.05).

To verify this conjecture, BALB/c mice were separated into a normal-dosage group, wherein mice were vaccinated using the fast vaccination strategy, and a triple-dosage group (**Figure 4A**). We employed CTL assay to analyze the antigen-specific cytolysis ability of splenocytes. At the 100:1 ratio of E:T, cytolysis of the fast vaccination group (11.83%) is higher than that of the triple-dose group (7.88%, *p* < 0.01) and that of the control group (5.61%, *p* <0.001). At 50:1 and 25:1 ratios, they have the same trend with the 100:1 ratio (**Figure 4B**). In ELISpot assay, splenocytes in the fast vaccination group secreted almost two-fold IFN-γ (353.5) of splenocytes in the triple-dose group (186) (*p* < 0.01, **Figure 4C**). According to CTL and ELISpot assay results, DNA vaccine administration under the fast immunization strategy elicited a stronger immune response than did the triple-dosage strategy (*p* < 0.01) (**Figures 4B, C**). The percentages of CD69^+^ in CD8^+^ cells and MDSCs (immunosuppressive cells) among splenocytes indicated that triple dosage increased MDSCs and decreased effector cells (*p* < 0.001) (**Figures 4D, E**). Further, triple dosage did not enhance the immune response and induced immune suppression instead. Thus, we aimed to confirm whether the amount of antigen was indeed associated with immune responses. Hence, the DNA vaccine dosage was reduced to 50 or 25 μg, administered under the fast immunization strategy, for comparison with the 100-μg dosage based on the resulting immune response (**Figure 4F**). The CTL assay revealed no significant differences between 100 and 50 μg (*p* > 0.05) vaccination. Both the 100- and 50-μg vaccine dosages induced greater CTL-mediated cytolysis than did the 25-μg dosage at an E/T ratio of 100:1 (*p* < 0.01, **Figure 4G**). ELISpot assay results confirmed no significant differences among the 100-, 50-, and 25-μg groups (*p* > 0.05, **Figure 4H**). These results suggested that dosage does not play an important role in the fast vaccination strategy, as opposed to the traditional strategy, wherein the immune response was dose-dependent (**Figure S1**).

The DNA vaccine dosage had little effect on the efficacy of the fast vaccination strategy as per the data presented in **Figure 4**. Triple dosage at once or a lower dose administered on three separate occasions elicited slightly different or similar immune responses. This strongly suggests that the migration of APCs to the injection site played the key role under fast vaccination.

### Expansion and Contraction Time Line of CD8^+^ T Cells After Fast Vaccination

**Figure 5:**
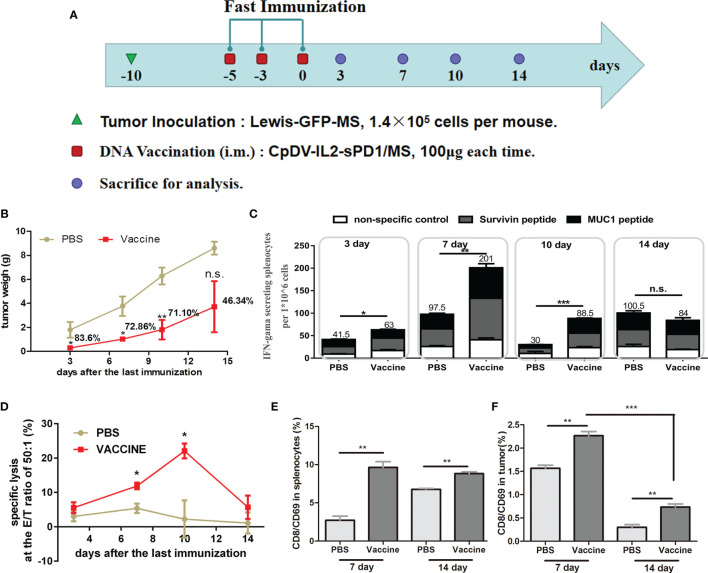
Anti-tumor effect and temporal immune response to the DNA vaccine in the fast immunization strategy. **(A)** Immunization regimen in tumor-bearing mice. Twenty-four C57BL/6 mice were separated into a PBS group (n = 12) and a vaccine group (n = 12), and 1.4 × 10^5^ Lewis-GFP-MS cells were subcutaneously injected into mice. Five days later, tumor volume reached approximately 50 mm^3^, and mice were vaccinated on days -5, -3, and 0 with 100 μg of CpDV-IL2-sPD1/MS (intramuscularly injected). Three mice per group were sacrificed on days 3, 7, 10, and 14. **(B)** Tumor weight. Tumor tissues were dissected and weighed. **(C)** ELISpot assay. Splenocytes were stimulated with peptides (survivin H2-D^b^ sequence: STFKNWPFL, final concentration: 10 μg/ml; MUC1 H2-D^b^ sequence: SAPDTRPAP, final concentration: 10 μg/ml). **(D)** Cytotoxic T lymphocyte (CTL) assay. Lewis and Lewis-GFP-MS cells were labeled with 0.5 and 5 μM carboxyfluorescein diacetate succinimidyl ester (CFSE), and mixed at a ratio of 1:1. The mixed Lewis and Lewis-GFP-MS cells were used as target cells to analyze the antigen-specific cytolytic ability of splenocytes *via* flow cytometry. Effector CD8^+^/CD69^+^ cells and suppressor cells were separated from splenocytes **(E)** and tumor tissue **(F)**. (*p < 0.05, **p < 0.01, ***p < 0.001, n.s., p > 0.05).

The above-described results highlighted the potential of fast vaccination as an alternative to the currently employed cancer vaccine administration regimens. As a new strategy, the expansion and contraction timeline of effector CD8^+^ T cells should be elucidated, and it would be useful when this fast DNA vaccination strategy is boosted with rAD vaccine in the following research. C57BL/6 mice were inoculated with Lewis-GFP-MS, vaccinated, and then sacrificed for analysis during the period of day 0 and day 14 after the last DNA vaccination (**Figure 5A**). To comprehensively reflect the antitumor effect, the tumor size was evaluated (**Figure 5B**). Three mice per group were sacrificed at each time point, and their tumors were dissected and weighed. When vaccines were administered under the fast immunization strategy, tumor growth was suppressed by 83.6% (*p* < 0.05, day 3), 72.86% (*p* < 0.05, day 7), 71.10% (*p* < 0.01, day 10), and 46.34% (*p* > 0.05, day 14) (**Figure 5B**). The function of antigen-specific IFN-γ secretion and cytolysis were also analyzed by ELISpot assay and CTL assay. ELISpot (**Figure 5C**) and CTL (**Figure 5D**) assays indicated that fast vaccination induced an effective immune response on the third day after the last vaccination, which persisted to the 10th day.

Tumor-infiltrating CD8^+^ T cells are thought to be the direct executor of killing tumor cells. Hence, we also analyzed the activated CD8^+^ T cells (CD8^+^/CD69^+^ cells) in both spleen (**Figure 5E**) and tumor tissue (**Figure 5F**) by FACS. At 7 and 14 days after the last vaccination, more CD8^+^/CD69^+^ cells were observed in both the spleen and tumors in the vaccine group than in the PBS group (*p* < 0.01, **Figures 5E, F**). Interestingly, CD8^+^/CD69^+^ cells showed an obvious reduction in tumor tissue at day 14 compared with day 7 in both groups (*p* < 0.001, **Figure 5F**).

### Fast Vaccination Suppressed Larger Tumors to a Greater Extent

**Figure 6:**
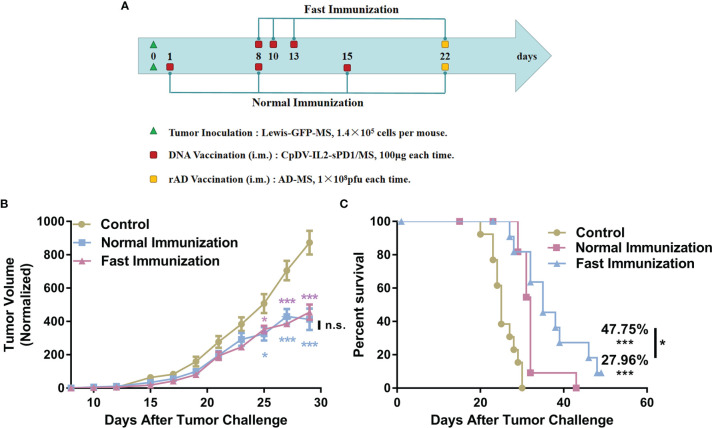
Improved antitumor effect of the DNA vaccine under the fast immunization strategy, even in larger tumors. **(A)** Tumor incubation and vaccination strategy regimen. Lewis-GFP-MS cells (1.4 × 10^5^) were injected subcutaneously into the right hind flanks of C57BL/6 mice. In the normal immunization group (n = 12), the DNA vaccine was administered 1 day later and at 1-week intervals thereafter. In the fast immunization group (n = 12), the DNA vaccine was administered on day 8, when the tumor volume reached approximately 50 mm^3^. The following two vaccinations were administered on days 10 and 13: 1 × 10^8^ pfu rAd on day 22 in both groups and PBS in control group mice (n = 13). **(B)** Tumor growth curves. Tumor volume measurements were taken beginning from day 8 after tumor cell inoculation. **(C)** Survival analysis was conducted by recording the death of tumor-bearing mice (*p < 0.05, ***p < 0.001, n.s., p > 0.05).

We sought to determine whether the faster and more potent immune response elicited under the fast immunization strategy would result in enhanced antitumor immunity. Further, due to the time-consuming immune response, the normal vaccination strategy has to initiate the treatment 1 day after tumor incubation in our previous research (29, 30, 32). In this part, the fast DNA vaccination strategy was challenged with initiating treatment till tumor mass was about 50-100 mm^3^. The first DNA vaccine was vaccinated 7 days later in the fast vaccination strategy group than that in the normal vaccination strategy group. The antitumor effect and survival time were analyzed (**Figure 6**). C57BL/6 mice in the normal vaccination group received the DNA vaccine on day 1 after tumor cell inoculation. Mice in the fast vaccination group received the DNA vaccine on day 8, when tumor volume reached approximately 50 mm^3^ (**Figure 6A**). Tumor size was suppressed by 47.82% in the fast vaccination group relative to that in the normal immunization group (52.68%). Even though the vaccine was administered 7 days later in the fast immunization group than in the normal immunization group, no significant difference in tumor growth suppression was observed between the two groups (*p* > 0.05) (**Figure 6B**). Moreover, the survival time of mice was prolonged by 47.45% in the fast immunization group (*p* < 0.001, vs. control group; p < 0.05 vs. normal group), and the survival time of mice was prolonged by 27.06% in the normal group (*p* < 0.001, vs. control group, **Figure 6C**). These data demonstrate that the fast immunization strategy induced stronger antitumor effects than the normal strategy did, even when vaccines were administered at a later stage.

### Safety

The safety evaluations tests of the fast DNA vaccination strategy was entrusted to Shandong Xinbo Pharmaceutical R&D, LED. Long-term toxicity test and acute toxicity tests were carried out on Balb/c mice and cynomolgus monkeys. These results showed that no obvious abnormality in weight gain was found in the Balb/c mice after treatment *via* rapid DNA vaccination strategy, and the major organs (such as heart, liver, kidney, lung, and spleen) of Balb/c mice and cynomolgus monkey showed no toxic effects (data not shown).

## Discussion

We developed a fast vaccination strategy, wherein a cancer DNA vaccine was intramuscularly administered on days 0, 2, and 5. This approach elicited a stronger immune response and exhibited enhanced antitumor efficacy. We also elucidated the potential mechanism underlying the effects of the fast vaccination strategy and observed an upregulation of CCL3, CCL5, and CCL19 as well as the recruitment of CD11c^+^ cells to the injection site. The timing of booster DNA vaccination was based on that of CD11c^+^ cell accumulation at the injection site. The presence of these cells most probably facilitated increased antigen presentation or cross-presentation. Several recent studies have reported the recruitment of DCs to the inflammation site caused by adjuvants or wounds, with these APCs playing a pivotal role in the initiation of subsequent immune responses (27–30). The fast vaccination strategy likely stimulated CD11c^+^ cell migration to increase the uptake of vaccine DNA as well as direct antigen uptake.

Our DNA vaccine was administrated intramuscularly instead of subcutaneously and elicited a stronger antitumor immune response than did the conventional 2-week-interval strategy. We analyzed the antitumor efficacy of fast vaccination in C57BL/6 mice subcutaneously inoculated with Lewis cells. We previously applied this strategy in a mouse model of breast cancer (4T1 breast cancer cell line, BALB/c mice) (42) as well as a pancreatic cancer C57BL/6 mouse model (Panc02 pancreatic cancer cell line, unpublished data). In all three cancer models, including both C57BL/6 and BALB/c mouse strains, fast vaccination showed consistent results, indicating that its beneficial effects are not strain-dependent and highlighting its potential as a superior alternative to conventional vaccination regimens.

Immunogens are a critical factor for immune response induction. However, according to our results, the amount of antigens did not influence the strength of the immune response elicited by the fast vaccination strategy. Under the fast vaccination regimen, the 25-μg DNA vaccine induced a similar immune response to that following the 100-μg DNA vaccine administration, which contrasted the dose-dependent effect observed under the normal vaccination strategy. Further, we administered a triple-dosage DNA vaccine to verify the relationship between antigen expression and the fast vaccination strategy. Our results indicated that the efficacy of fast vaccination was not dependent on the DNA vaccine dose. This observation is in agreement with the report by Schumacher et al., who suggested that antigen expression is not the key factor underlying the efficacy of fast vaccination *via* intramuscular injection (7). We attribute this enhanced immune response to the accumulation of CD11c^+^ APCs at the injection site and the immunoadjuvants in our vaccine. In future research, the role of immunoadjuvants in upregulating chemokines and recruiting APCs should be explored in greater detail.

Heterologous prime-boost immunization is used to enhance the immune response induced by DNA vaccines. In our previous research, we found that rAd boost enhanced the efficacy of CpDV-IL2-sPD1/MS under the normal immunization strategy. Hence, we applied the rAd boost under fast immunization and compared it with the normal strategy. We also analyzed the cell subtypes implicated in the immune response after fast vaccination.

Importantly, the fast vaccination strategy not only represents a time-saving option but also exhibited antitumor efficacy following vaccine administration at a later point of disease progression as opposed to the traditional strategy. We attribute this outcome to the shorter vaccination regimen and the more rapid peaking of the immune response. Research has shown that tumors establish a tumor-associated microenvironment as the disease progresses in order to evade immune surveillance, chemotherapy, and radiotherapy (43–45). Therefore, earlier immune response induction allows for greater tumor cell killing.

Additionally, the rapid vaccination strategy has the advantage in combined therapies. Due to the heterogeneity of cancer, a single treatment is hard to wipe out all the tumor cells. Therefore, cancer vaccines are always used together with chemotherapy or radiotherapy. It is difficult to determine the effects of these chemotherapy or radiotherapy on immunotherapy during combination treatment. Sometimes, chemotherapy or radiotherapy killed the suppressed immune cells, and sometimes they killed effector immune cells. The fast vaccination strategy could be applied between the courses of chemotherapy and avoiding decreasing the effector immune response.

Moreover, according to the safety evaluations tests, the rapid DNA vaccination strategy showed an excellent safety. Chen et al. found that the immune system, including IL-22, was closely related to the recovery of kidney and liver (46–48). These results inspire us that DNA cancer vaccine is an effect cancer treatment with good safety.

In the current study, the cancer DNA vaccine CpDV-IL2-sPD1/MS was administered under a fast vaccination strategy and demonstrated enhanced efficacy relative to the conventional vaccination strategy. Future research should explore this approach with other vaccines and elucidate the molecular mechanism of DC recruitment within muscle tissue.

## Data Availability Statement

The original contributions presented in the study are included in the article/**Supplementary Material**. Further inquiries can be directed to the corresponding authors.

## Ethics Statement

The animal study was reviewed and approved by the Ethical Committee for the Use of Laboratory Animals at Jilin University.

## Author Contributions

CL: conceptualization, data curation, formal analysis, investigation, visualization, writing—original draft, and funding acquisition. XC and YW: conceptualization and formal analysis. QG, YX, FG, JG, LD, and YZ: investigation. HW, BY, and JW: methodology. HZ: conceptualization, funding acquisition, project administration, supervision, writing—review, and editing. XY: project administration, supervision, and validation. WK: funding acquisition, project administration, and resources. All authors contributed to the article and approved the submitted version.

## Funding

This study was supported by Projects of Science and Technology Department of Jilin Province, China [no. 20210101249JC], Key R & D Projects of Science and Technology Department of Jilin Province, China [no. 20180201001YY], Major Projects of Science and Technology Innovation in Changchun City, China [no. 17YJ002], the Specialized Research Fund for the National Natural Science Foundation of China [no. 31300765], the Jilin Province Science and Technology Development Program, China [no. 20160519018JH], International Cooperation Projects of Science and Technology Department of Jilin Province, China [no. 20190701061GH], and Youth Learner Fund for the National Natural Science Foundation of China [no. 81601445]. We declare all sources of funding received for the research being submitted.

## Conflict of Interest

The authors declare that the research was conducted in the absence of any commercial or financial relationships that could be construed as a potential conflict of interest.

## Publisher’s Note

All claims expressed in this article are solely those of the authors and do not necessarily represent those of their affiliated organizations, or those of the publisher, the editors and the reviewers. Any product that may be evaluated in this article, or claim that may be made by its manufacturer, is not guaranteed or endorsed by the publisher.

## Supplementary Material

Supplementary Figure 1 Immune response induced by the DNA vaccine under the normal vaccination strategy depends on the vaccine dosage. **(A)** Immunization regimen. Twenty BALB/c mice were divided into four groups (n=5). DNA vaccine doses included 25, 50, and 100 μg in the respective groups. Vaccines were administered intramuscularly three times at two-week intervals. Recombinant adenovirus (rAd) (1×10^8^ pfu in 100 μl per mouse) was administered two weeks after administration of the last DNA vaccine dose **(B)** ELISpot assay. Ten days after rAd vaccination, all mice were sacrificed to perform the ELISpot assay. Splenocytes were incubated with hSurvivin peptide (H2-K^d^ sequence: AFLSVKKQF, final concentration: 10 µg/mL) and hMUC1 peptide (H2-K^d^ sequence: APDTRPAPG, final concentration: 10 µg/mL), and the secretion of IFN-γ per million splenocytes (ELISpot assay) was determined. (n.s. p > 0.05, *p < 0.05, **p < 0.01, ***p < 0.001).

## Abbreviations

APC, antigen-presenting cell; DC, dendritic cell; rAd, recombinant adenovirus; qRT-PCR, quantitative real-time PCR; CTL, cytotoxic T lymphocyte; CFSE, carboxyfluorescein diacetate succinimidyl ester; FACS, flow cytometry; MDSCs, myeloid-derived suppressor cells; iDC, immature DC; mDC, mature DC.
