# Supplementary figures and images for: Fast DNA Vaccination Strategy Elicits a Stronger Immune Response Dependent on CD8+CD11c+ Cell Accumulation
Source: Front Oncol. 2021 Dec 7;11:752444. doi: 10.3389/fonc.2021.752444 (PMC8691261; doi:10.3389/fonc.2021.752444)

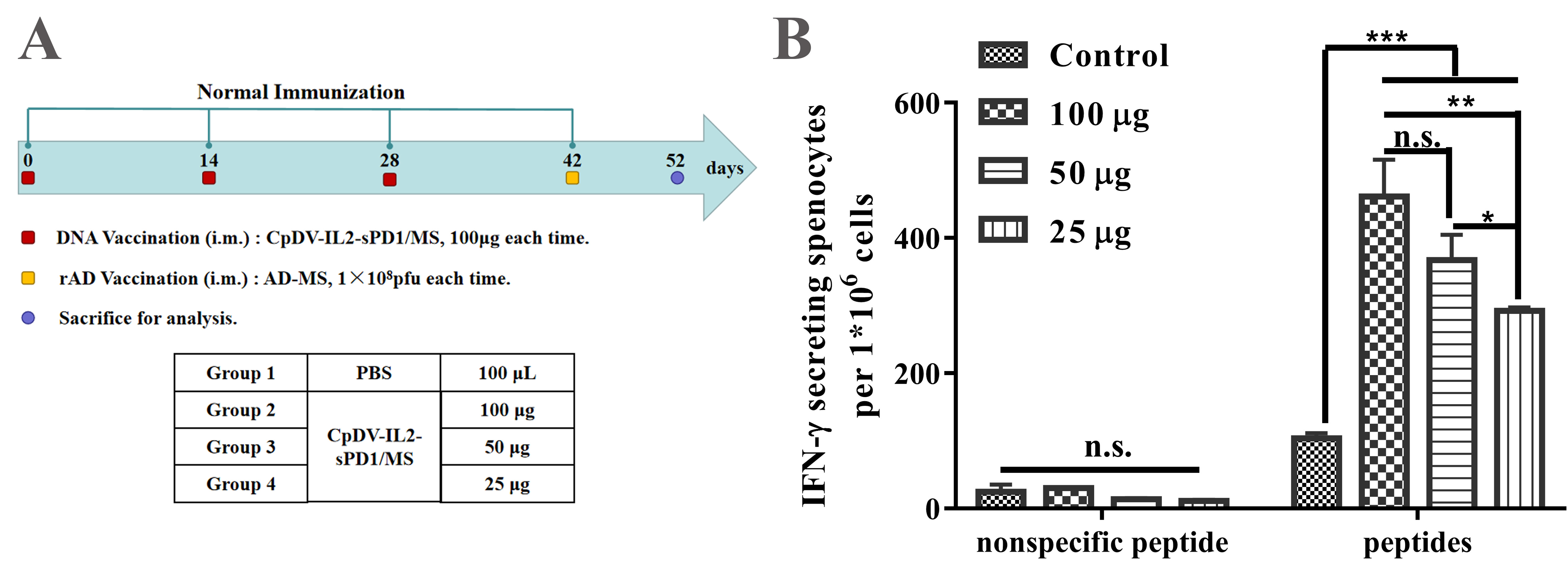

Supplement: Supplementary Figure 1 — Immune response induced by the DNA vaccine under the normal vaccination strategy depends on the vaccine dosage. (A) Immunization regimen. Twenty BALB/c mice were divided into four groups (n=5). DNA vaccine doses included 25, 50, and 100 μg in the respective groups. Vaccines were administered intramuscularly three times at two-week intervals. Recombinant adenovirus (rAd) (1×10^8^ pfu in 100 μl per mouse) was administered two weeks after administration of the last DNA vaccine dose (B) ELISpot assay. Ten days after rAd vaccination, all mice were sacrificed to perform the ELISpot assay. Splenocytes were incubated with hSurvivin peptide (H2-Kd sequence: AFLSVKKQF, final concentration: 10 µg/mL) and hMUC1 peptide (H2-Kd sequence: APDTRPAPG, final concentration: 10 µg/mL), and the secretion of IFN-γ per million splenocytes (ELISpot assay) was determined. (n.s. p > 0.05, *p < 0.05, **p < 0.01, ***p < 0.001). [file Image_1.jpeg]
